# Investigation of the Optical Properties of Indium Tin Oxide Thin Films by Double Integration Sphere Combined with the Numerical IAD Method

**DOI:** 10.3390/ma16041425

**Published:** 2023-02-08

**Authors:** Alejandro Toral-Lopez, María M. Pérez, Ana Belen Rodríguez-Águila, Juan C. Cardona, Ana M. Ionescu, Andres Godoy

**Affiliations:** 1 Pervasive Electronics Advanced Research Laboratory (PEARL), Department of Electronics and Computer Technology, University of Granada, 18071 Granada, Spain; 2 Laboratory of Biomaterials Optics, Department of Optics, University of Granada, 18071 Granada, Spain

**Keywords:** ITO, double integration sphere, IAD, transmittance, absorption, scattering, mechanical stress

## Abstract

Transparent conductive electrodes have become essential components of numerous optoelectronic devices. However, their optical properties are typically characterized by the direct transmittance achieved by making use of spectrophotometers, avoiding an in-depth knowledge of the processes involved in radiation attenuation. A different procedure based on the Double Integration Sphere combined with the numerical Inverse Adding-Doubling (IAD) method is employed in this work to provide a comprehensive description of the physical processes limiting the light transmittance in commercial indium tin oxide (ITO) deposited on flexible PET samples, highlighting the noticeable contribution of light scattering on the total extinction of radiation. Moreover, harnessing their flexibility, the samples were subjected to different mechanical stresses to assess their impact on the material’s optical and electrical properties.

## 1. Introduction

Transparent conductive electrodes are receiving increasing attention as they play a crucial role in multiple optoelectronic devices such as touch panels [1,2], e-paper [3], solar cells [4,5], and light-emitting diodes [6]. To optimize their performance, it becomes mandatory to use materials that simultaneously combine high electrical conductivity with high optical transparency, and ideally, to achieve greater versatility, they should also be flexible. One of the drawbacks of using transparent oxide films is that they are brittle by nature and therewith limit the flexibility of the device [7]. Tin (Sn) doped In_2_O_3_ films, familiarly known as indium tin oxide (ITO), have been widely studied and employed as transparent conducting electrodes (TCEs) for the last decades. This material has proven unique characteristics such as reduced resistivity (as low as 1 mΩ·cm), high optical transmittance in the visible wavelength range (>80%), and good adhesion to the substrate [8,9,10]. However, there is an unavoidable trade-off between the electrical resistivity and the optical transparency, as to increase the electrical conductivity, a thicker film is necessary and that, in turn, reduces the material’s transparency [11].

A number of different techniques such as spray pyrolysis [12], electron beam evaporation, colloidal synthesis [13], pulsed laser deposition [14], thermal evaporation, screen printing, ion-assisted plasma evaporation [15], sol-gel process [16], chemical/physical vapor deposition [17], etc., have been used to deposit ITO thin films. Besides all these methods, spin coating [18] and magnetron sputtering [19] are the most common techniques since they offer the optimum compromise to achieve high-quality films over wide area substrates. The temperature employed in the fabrication process determines the microstructure of the material as low temperatures yield amorphous materials while the use of a high-temperature methodology gives rise to polycrystalline ITO [20]. In addition, it has clearly been demonstrated that crystalline materials provide better performance than their amorphous counterpart [20,21]. However, the maximum temperature of the fabrication process could be limited by the substrate where ITO is deposited. On the other hand, for flexible applications (e.g., wearables), the ITO microstructure could be altered by excessive mechanical stress due to its inherent brittle nature. Kim et al. [22] demonstrated that the electrical resistivity of a ZnSnO (20 nm)/Ag (10 nm)/ITO (30 nm) multilayer electrode on a polyethylene terephthalate (PET) substrate was hardly affected by cyclic bending, whereas a thicker ITO reference film showed a noticeable resistivity increase after several bending cycles. However, the effect of the bending stress on the optical transparency of the ITO has not been studied to date.

Several studies in the literature [18,22,23,24] have systematically determined and analyzed the optical properties of TCEs, and most of them have been mainly focused on the evaluation of the direct optical transmittance and/or reflectance in the visible and/or near-infrared wavelengths ranges making use of spectrometer measurements. Mahmouidi et al. compared in [25] spectral transmittance values of different TCEs such as ITO, carbon nanotubes, Ag nanowires, organic materials, metal nanoparticles, aluminum-doped zinc oxide, and graphene. All of them exhibited transmittance values within the range of 82% to 90% (only graphene showed a value higher than 90%). However, this type of measurement does not provide conclusive information to get a deep understanding of light interaction within the material. This analysis of the optical properties of materials is commonly described in terms of the absorption coefficient, $\mu_{a}$, and the scattering coefficient, $\mu_{s}$, according to the radiative-transport equation [26]. The determination of these coefficients provides the contribution of light scattering and absorption to radiation extinction (light transmittance).

The complexity of the material structure hinders the analytical solution of this equation, requiring the use of numerical iterative procedures such as the Inverse Adding-Doubling (IAD) method [27]. This procedure must be applied in conjunction with reflection and transmission measurements performed with an integrating sphere or a double integrating sphere (DIS) system, to estimate the main optical properties of the media represented by their scattering and absorption coefficients. Nevertheless, up to now, this methodology has been mainly used for the determination of the optical properties of organic biological tissues [28], and not for the investigation of the absorption and scattering characteristics of inorganic TCEs.

One of the main advantages of the DIS system versus other procedures such as the Kubelka-Munk method [29,30] and systems of reflection and transmission measurements, is that the total reflectance and total transmittance are acquired simultaneously with the sample in the same position, under the same illumination conditions and mechanical constraints. Thus, the use of the DIS measurement system is favored due to its improved accuracy [31]. Moreover, according to the numerous studies that make use of it, some authors [31,32] describe the DIS measurement techniques as the “golden standard” method for determining optical properties.

Hence, this work aims to investigate optical properties such as the absorption and the scattering of incident light on ITO, considered the most representative material for transparent conductive electrodes, making use of the numerical IAD method combined with a DIS experimental setup. Additionally, the effect of flexural stress on the light transmittance of the ITO films will be assessed for its potential use in flexible electronics.

## 2. Materials and Methods

Of the whole family of TCEs, we selected indium tin oxide (ITO) as the most commonly used display application. In particular, we considered ITO deposited on polyethylene terephthalate (PET) films, hereinafter referred to as ITO-PET, as raw materials in this work. ITO-PET is commonly used in the fabrication of flexible organic light-emitting diodes (OLEDs) [33], and also as a reference anode in conventional flexible polymer-based organic solar cells (OSCs) [34]. For this study, we have employed commercial samples provided by two vendors [35,36,37,38] whose main properties are shown in Table 1.

**Figure 1:**
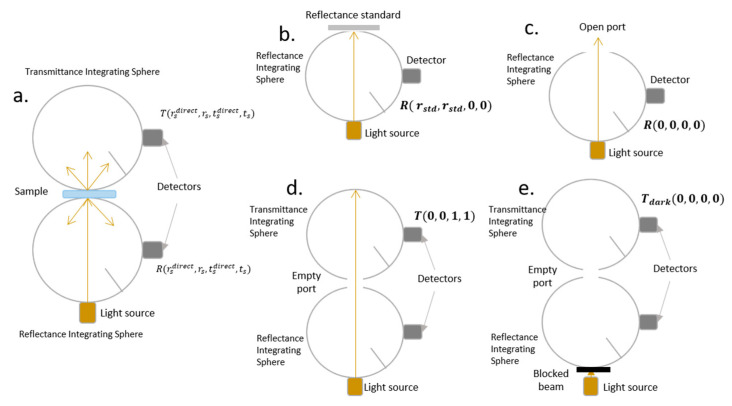
Schematic representation of the experimental setup depicting the double integrating sphere (DIS). (**a**) reflection and transmission measurements of the sample; (**b**) reflection measurement of the reflectance standard; (**c**) dark measurement for the reflection setup; (**d**) measurement with an empty port corresponding to 100% transmission and (**e**) dark measurement for the transmission setup.

It is a common practice to describe the optical properties of the ITO samples in terms of their direct transmittance measured with a UV/visible spectrometer. However, this procedure provides limited information on the physical processes determining the optical performance of any material. Thus, to assess the optical properties of the ITO-PET samples, the Inverse Adding-Doubling (IAD) method [27] was used in conjunction with the experimental setup depicted in Figure 1 for measuring the total diffuse reflectance and total diffuse transmittance of light transmitted through the samples under study.

The double integrating sphere (DIS) measuring system [32], as represented in Figure 1a, consisted of two spheres with the sample placed between them. Briefly, two 60-mm-diameter integrating spheres (Oriel, model 70674, Stratford, CT, USA) each one with a 3-mm-diameter detector port, a 12.5-mm-diameter sample port with a baffle between ports, and also a 12.5-mm-diameter entrance port, were used for total reflection and total transmission measurements. A white light source (240–1100 nm, Thorlbas, Germany) connected to the double integrating sphere system by an optic fiber (M92L01, Φ = 200 µm, 0.22 NA) was used as a light source. The ITO-PET samples were sandwiched between two optical borosilicate glass slides (1.1 mm thickness).

The total reflection measurements were carried out three times for each sample and, in all the cases, the sample’s lateral size exceeded the diameter of the sphere sample port. Reflection measurements of our samples were referenced to a 98% optopolymer reflectance standard (OPST3-C, Optopolymer, Germany, Figure 1b) and a dark measurement, where the sample port was empty (Figure 1c). The total diffuse reflectance ($M_{R}$), was calculated as a percentage according to the following expression:

(1) $$M_{R}=r_{std}\frac{R\left(r_{s}^{direct},\;r_{s},t_{s}^{direct},\;t_{s}\right)-R\left(0,0,0,0\right)}{R\left(r_{std},\;r_{std},0,0\right)-R\left(0,0,0,0\right)}\;$$

where $r_{std}$ is the reflection of the reflectance standard, $R\left(r_{s}^{direct},\;r_{s},t_{s}^{direct},\;t_{s}\right)$ is the reflection measurement of the sample of interest, $R\left(r_{std},\;r_{std},0,\;0\right)$ is the reflection measurement of the reflectance standard, and R(0,0,0,0) is the dark measurement (sample port empty).

The total diffuse transmittance was measured under the same setup conditions, making use of the DIS setup with collimated light. Transmittance measurements of the samples were referenced to 100% with the illuminating source activated and the sample port empty (Figure 1d) and a dark measurement was carried out with an open port without any illumination source (Figure 1e). The total diffuse transmittance was calculated, also as a percentage, using the following expression:

(2) $$M_{T}=\frac{T\left(r_{s}^{direct},\;r_{s},t_{s}^{direct},\;t_{s}\right)-T_{dark}\left(0,0,0,0\right)}{T\left(0,0,\;1,1\right)-T_{dark}\left(0,0,0,0\right)}\;$$

where $T\left(r_{s}^{direct},\;r_{s},t_{s}^{direct},\;t_{s}\right)$ is the transmission measurement of the sample of interest, T(0,0, 1,1) is the transmission measurement of the empty sample port, and $T_{dark}\left(0,0,0,0\right)$ is the dark measurement with an open port without any illumination.

From the diffuse reflectance ($M_{R}$) and diffuse transmittance ($M_{T}$) measurements, the absorption coefficient ($\mu_{a}$) and the reduced scattering coefficient ($(\mu_{s}^{’}=\mu_{s}\left(1-g\right)$, where g is the scattering anisotropy factor) were determined using the IAD method [39]. With values of ***µ_a_*** and $\mu_{s}^{’}$ obtained numerically, we can calculate new values of $M_{R}$ and $M_{T}$ that will be compared with the measured ones. According to the IAD algorithm [27], this process is iterated until the calculated and experimentally obtained values of $M_{R}$ and $M_{T}$ are within a specified tolerance (for this work the tolerance default value was set to 0.01%). A flowchart of the IAD algorithm is shown and explained in Figure S1 of the Supplementary Information.

To solve the inverse problem, knowledge of the refractive index of the sample is required. Changes in the refractive index over the range of wavelengths employed in the present study were assumed negligible and, therefore, we consider the refractive index ***n*** = 1.827 for **λ** = 590 nm as the mean value for the visible range [40]. To determine the scattering anisotropy factor, we employed a customized goniometric optical setup (Figure S2) to measure the angular distribution of scattered light within the angular range of 0–175° [41]. The g values obtained are indicated in Table 2:

The key role played by the absorption and the scattering processes in the extinction of the light when passing through the analyzed sample, is evaluated with the albedo coefficient (a) that can be calculated in terms of ***µ_a_*** and $\mu_{s}^{’}$, according to the following equation [42]:

(3) $$a=\frac{\mu_{S}^{’}}{\mu_{S}^{’}+\mu_{a}}\;$$

In addition, to determine the level of similarity regarding the spectral behavior of different optical magnitudes, the Variance Accounting For (VAF) coefficient with Cauchy-Schwarz inequality is used. This statistical parameter is defined as follows [43]:

(4) $$VAF=\frac{{\left(\sum_{k=380}^{780}a_{k}.b_{k}\right)}^{2}}{\left(\sum_{k=380}^{780}a_{k}^{2}\right)\left(\sum_{k=380}^{780}b_{k}^{2}\right)}$$

where $a_{k}$ is the spectral value of $M_{R}$, $M_{T}$, $\mu_{a}$ and $\mu_{s}^{’}$ (for wavelengths from 380 to 780 nm) and $b_{k}$ is the equivalent parameter for the measurement to be compared. The closer this coefficient gets to unity (100%), the more similar the two curves become.

**Figure 2:**
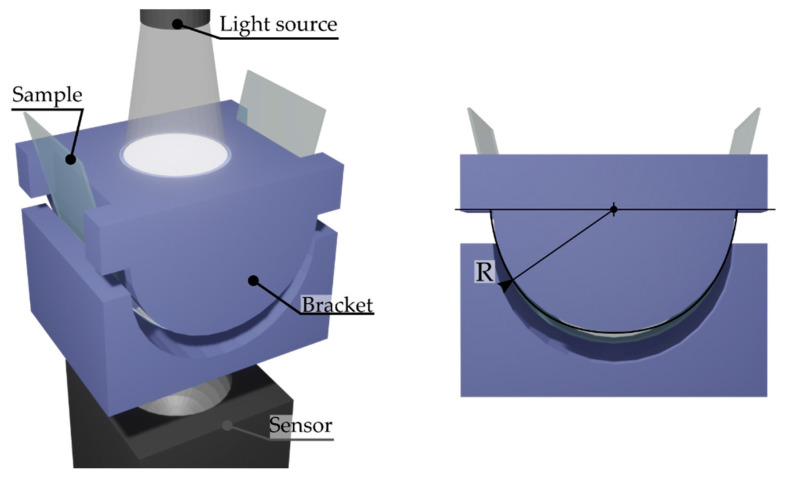
Schematic representation of the experimental setup employed to measure the different strained samples.

Finally, to investigate the effect of mechanical flexibility on light transmittance, the direct transmittance (T%) of radiation propagating throughout the visible spectral range was calculated from the incident and transmitted intensities measured using a spectrometer (Thorlabs CCS200/M, 200–1000 nm) with a spectral light source (BDS130 Deuterium/Tungsten, 190–2500 nm) as:

(5) $$T=\frac{I}{I_{0}}\;$$

where *I*_0_ is the incident light intensity, and *I* is the transmitted light intensity. Transmittance was a measure for tensile and compressive strain using customized circular brackets, providing four different radii of curvature, 37.5, 75, 150, and 300 mm, respectively as shown in Figure 2. For the sake of completeness, the resistance of the strained samples was also measured for each curvature. The double-integrating sphere measuring system does not allow the coupling of customized brackets, therefore, the ITO-PET samples under bending stress were measured only for their direct transmittance.

## 3. Results and Discussion

**Figure 3:**
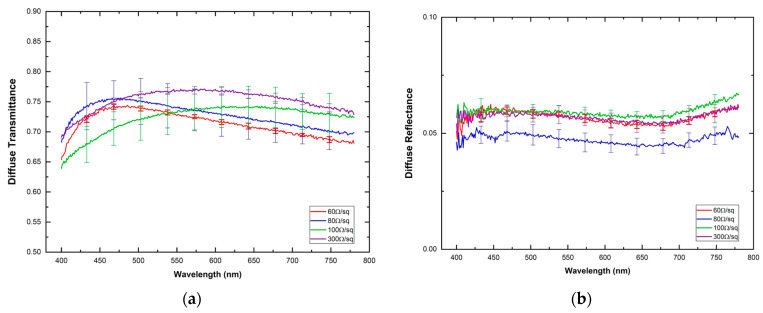
Spectral diffuse reflectance (**a**) and diffuse transmittance (**b**) for each one of the ITO-PET electrodes. Solid lines correspond to the mean value extracted from the measurements and their standard deviation is also indicated as vertical lines.

Figure 3 depicts the spectral diffuse reflectance $\left(M_{R}\right)$ and diffuse transmittance $\left(M_{T}\right)$ of the four ITO-PET samples in the visible range (400–780 nm). The diffuse reflectance distributions of the ITO-PET electrodes display a similar spectral behavior for all the surface resistances analyzed (VAF > 99.0%), with values ranging between 6.7% (ITO-PET 100 Ω/sq at 777 nm), and 4.3% (ITO-PET 80 Ω/sq at 400 nm). Furthermore, for the whole wavelength range, the reflectance shows a lower value for the 80 Ω/sq samples, as this material was provided by a different provider. In general, spectral reflectance increases for the initial range from 400 nm to 450 nm, keeps a gentle reduction in the central portion of the visible spectrum, and then shows a slight upswing above 675 nm. On the other hand, the diffuse transmittance increases from 400 nm to 500 nm. For higher wavelengths, the transmittance remains approximately constant for the 100 Ω/sq and 300 Ω/sq ITO-PET electrodes, while for the lower resistance values, 60 Ω/sq and 80 Ω/sq, $M_{T}$ displays a smooth reduction. The $M_{T}$ values ranged between 64.0% (ITO-PET 100 Ω/sq at 400 nm) and 77.1% (ITO-PET 300 Ω/sq at 546 nm). Also, as for $M_{R}$, the spectral transmittance showed similar spectral behavior for all the surface resistivity analyzed. This conclusion was statistically corroborated by a VAF value of 99.0%.

**Figure 4:**
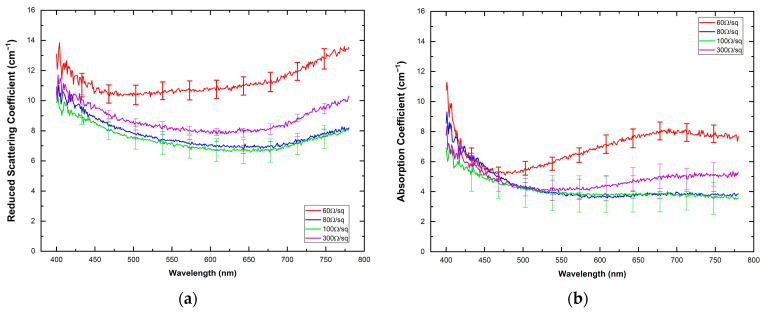
Reduced scattering coefficient (**a**) and absorption coefficient (**b**) of the ITO-PET electrodes. Solid lines correspond to the mean value extracted from the measurements and their corresponding standard deviation is also indicated as vertical lines.

Figure 4a,b shows the spectral distribution of the scattering and absorption coefficients for the ITO-PET electrodes evaluated. The spectral behavior of the reduced scattering coefficient, $\mu_{s}^{’}$, displayed higher values, ranging between 7 cm^−1^ (ITO-PET 100 Ω/sq at 652 nm) and 14 cm^−1^ (ITO-PET 60 Ω/sq at 774 nm) than the absorption coefficient, $\mu_{a}$, 4 cm^−1^ (ITO-PET 80 Ω/sq at 598 nm) and 8 cm^−1^ (ITO-PET 60 Ω/sq at 689 nm) for the visible range analyzed in this study. The scattering coefficient of the analyzed ITO-PET showed similar spectral behavior for high and low resistivity (VAF > 99.0%). The highest values of scattering and absorption for the ITO-PET of 60 Ω/sq explain the lower transmittance of this electrode, a behavior related to the higher ITO coating thickness (130 nm) [44].

**Figure 5:**
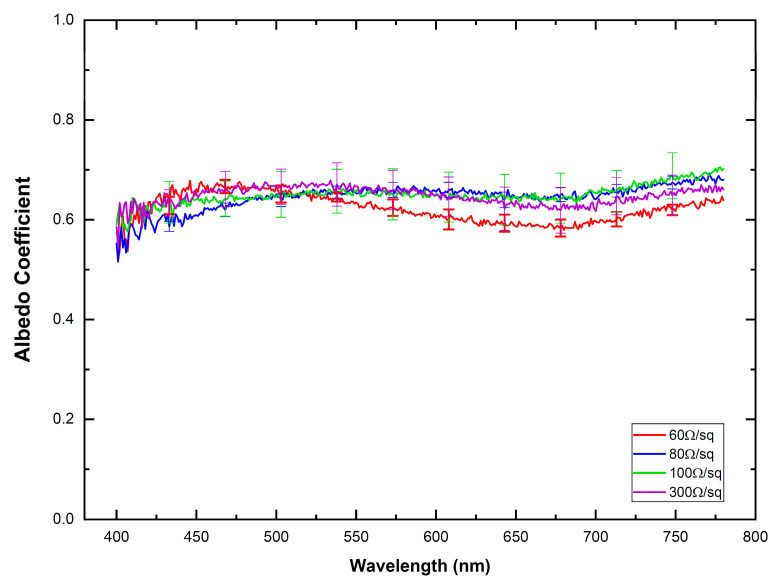
Spectral distribution of albedo coefficient for the different ITO-PET electrodes. Solid lines correspond to the mean value extracted from the measurements and their standard deviation is also indicated as vertical lines.

According to Figure 4, scattering is the most relevant optical extinction phenomenon that occurs when light interacts with the ITO-PET samples. This conclusion is corroborated by the albedo coefficient shown in Figure 5, with values above 0.6 regardless of the electrode resistivity. The high values of the anisotropy coefficient, g, obtained in this study (Table 2), close to one for all the ITO-PET electrodes highlight the anisotropy of this material explaining the prevalence of the scattering over the absorption for the light transmitted through these materials. This optical behavior follows a trend similar to that observed in translucent biological tissues [28,42] and dental zirconia [45], where the scattering coefficient is usually higher than the absorption. Anisotropic materials and materials with complex structures display higher scattering properties. Although the present study does not contemplate the evaluation of the structure and morphology of ITO thin films, previous studies [46,47] determined these characteristics, confirming their complex structure. 

**Figure 6:**
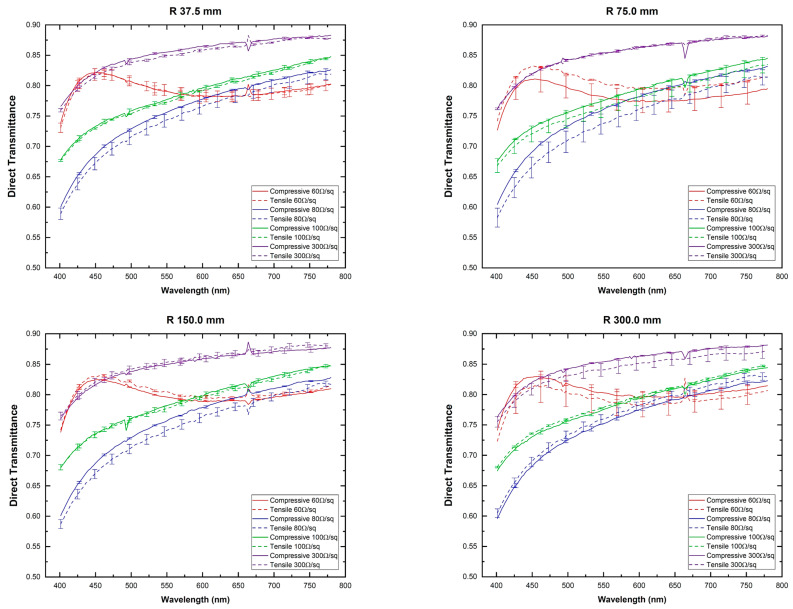
Direct transmittance was measured for different ITO-PET samples and four radii of curvature.

To assess the impact of mechanical stress on the transmittance (T%) and resistivity of the electrodes, both compressive (C) and tensile (T) strains corresponding to four different radii of curvature: 37.5 mm, 75 mm, 150 mm, and 300 mm were applied, and the direct transmittance measured as shown in Figure 6.

These results show that the analyzed samples present the same spectral behavior for both, compressive and tensile strain (VAF > 99.1%) for all ITO-PET samples and all the curvatures under study. Direct transmittance increases rapidly for increasing wavelength up to 500 nm from which a gentle increase is observed up to 780 nm. The 60Ω/sq sample shows a different behavior with a peak near 450 nm and a smooth variation up to 780 nm, a feature that agrees with previous studies on MOCVD-ITO grown on sapphire [48]. This behavior can be explained by attending to the absorption coefficient shown by this material in Figure 4, as the obtained transmittance follows a reverse trend with the absorption coefficient.

It has to be highlighted that direct transmittance does not show a clear modification as a consequence of the substrate curvature as all of them depict similar behavior. According to the manufacturer indications [35,36,37,38], a radius of curvature of up to 75 mm would not damage the ITO coating, and in our case, we have not observed significant modifications of both, optical and electrical performance when halving this radius to 37.5 mm.

Along with the optical characterization, the surface resistivity of the ITO-PET samples was measured (Keysight E4980AL Precision LCR Meter and Keysight 16089B Kelvin Clip Leads) for the aforementioned radius, and the obtained values are summarized in Table 3.

The fluctuations in the data are due to variability among the samples rather than the impact of the mechanical deformation of the experiments as a definite trend is not observed. Moreover, these measurements were repeated two months after the initial ones without any noticeable variation. These results prove that the ITO-PET samples are not degraded when mechanical strain is applied up to a minimum radius of 37.5 mm, providing a technological option for conformal electrodes in slightly curved surfaces.

## 4. Conclusions

This work provides a methodology to carry out a comprehensive optical characterization of transparent conductive electrodes (TCEs) that goes well beyond the usual measurements of direct transmissions making use of spectrometers. From this optical characterization, we can evaluate the scattering and the absorption coefficients of different ITO-PET samples, the TCE was chosen for this study, as they give insight into the physical mechanisms limiting the radiation transmittance through the material of interest. It can be concluded that scattering is the most relevant optical extinction phenomenon that occurs when light interacts with the ITO-PET samples, a conclusion corroborated by the albedo coefficient. This technique can be applied to arbitrary TCEs and relate the values attained for both coefficients with the processes followed in their fabrication. Moreover, we have also analyzed the impact of mechanical stress on the optical and electrical properties of the ITO-PET with different electrical resistance, up to a curvature radius of 37.5 mm without observing a noticeable degradation.

## Figures and Tables

**Table 1:** Properties of the raw materials employed in the study, as provided by the manufacturer.

	60 Ω/sq	80 Ω/sq	100 Ω/sq	300 Ω/sq
Reference	639303 [35]	[36]	639281 [37]	749796 [38]
Thickness	0.13 mm	0.175 mm	0.13 mm	0.13 mm
ITO Thickness	130 nm	23 nm	72 nm	24 nm
Transmittance (≅550 nm)	≥78%	≥88%	≥78%	≥78%

**Table 2:** Scattering anisotropy factor (g) for the different samples measured.

$R_{S}(Ω/\mathrm{sq})$	60 Ω/sq	80 Ω/sq	100 Ω/sq	300 Ω/sq
g	0.985 ± 0.011	0.987 ± 0.016	0.989 ± 0.021	0.989 ± 0.014

**Table 3:** Results obtained from the measurement of the surface resistivity (Ω/sq) of the ITO-PET samples under different radii of curvature. Measurements are expressed as (Ω/sq) and dispersion as %.

	Compressive Strain				Tensile Strain			
	300 mm	150 mm	75 mm	37.5 mm	300 mm	150 mm	75 mm	37.5 mm
60 Ω/sq	141 ± 9	137 ± 3	172 ± 10	162 ± 17	150 ± 5	153 ± 6	151 ± 6	167 ± 10
80 Ω/sq	557 ± 1	645 ± 5	624 ± 3	593 ± 4	600 ± 3	653 ± 3	559 ± 1	639 ± 4
100 Ω/sq	291 ± 10	299 ± 2	280 ± 3	282 ± 4	284 ± 4	265 ± 8	279 ± 1	289 ± 6
300 Ω/sq	1080 ± 1	1285 ± 3	1165 ± 3	1157 ± 8	1095 ± 5	1012 ± 4	1411 ± 2	1238 ± 15

## Data Availability

Not applicable.

## Supplementary Materials

The following supporting information can be downloaded at: https://www.mdpi.com/article/10.3390/ma16041425/s1, Figure S1. Flowchart representing the Inverse Doubling-Adding algorithm; Figure S2. Schematic representation of the experimental setup employed to measure the scattering anisotropy factor.
