# Supplementary material for: Investigation of the Optical Properties of Indium Tin Oxide Thin Films by Double Integration Sphere Combined with the Numerical IAD Method
Source: Materials (Basel). 2023 Feb 8;16(4):1425. doi: 10.3390/ma16041425 (PMC9967440; doi:10.3390/ma16041425)
Supplement: Supplementary file 1 [file materials-16-01425-s001.zip › materials-2139656-supplementary.pdf]

materials

Supplementary Materials

Investigation of the Optical Properties of Indium Tin Oxide Thin Films by Double Integration Sphere Combined with the Numerical IAD Method

Alejandro Toral-Lopez [1], María M. Pérez [2,*], Ana Belen Rodríguez-Águila [2], Juan C. Cardona [2], Ana M. Ionescu [2] and Andres Godoy [1]

P1 Pervasive Electronics Advanced Research Laboratory (PEARL), Department of Electronics and Computer Technology, University of Granada, 18071 Granada, Spain; atoral@ugr.es (A.T.-L.); agodoy@ugr.es (A.G.)

2 Laboratory of Biomaterials Optics, Department of Optics, University of Granada, 18071 Granada, Spain; anarodriguezaguila@correo.ugr.es (A.B.R.-Á.); cardona@ugr.es (J.C.C.); anaionescu@ugr.es (A.M.I.)

* Correspondence: mmperez@ugr.es

Citation: Toral-Lopez, A.; Pérez, M.M.; Rodríguez-Águila, A.B.; Cardona, J.C.; Ionescu, A.M.; Godoy, A. Investigation of the Optical Properties of Indium Tin Oxide Thin Films by Double Integration Sphere Combined with the Numerical IAD Method. *Materials* **2023**, *15*, x. https://doi.org/10.3390/xxxxx

Academic Editor(s): Dimitra Vernardou; Chunhua Chen

Received: 19 December 2022
Revised: 24 January 2023
Accepted: 3 February 2023
Published: date

Copyright: © 2023 by the authors. Licensee MDPI, Basel, Switzerland. This article is an open access article distributed under the terms and conditions of the Creative Commons Attribution (CC BY) license (https://creativecommons.org/licenses/by/4.0/).

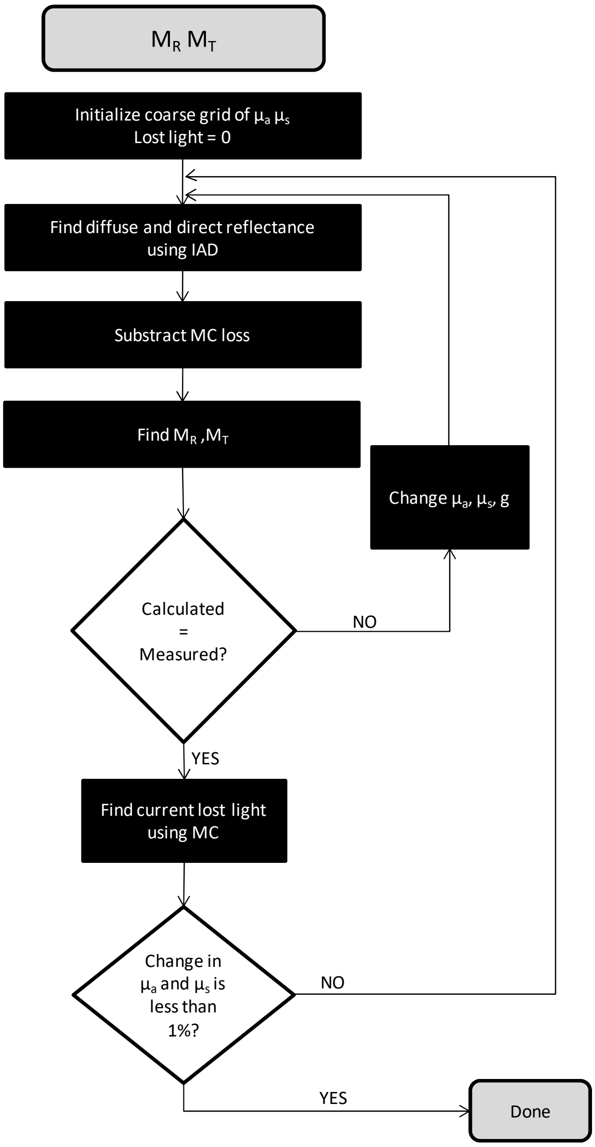

Figure S1. Flowchart representing the Inverse Doubling-Adding algorithm employed to estimate the optical parameters μ_a and μ_s' .

Initially, light lost through the ITO sample edge is fixed at zero and the program automatically generates a coarse grid to calculate reflectance and transmittance values with stipulated absorption coefficient, reduced scattering coefficient, and scattering anisotropy factor (**g**).The diffuse reflectance and the diffuse transmittance are calculated by the inverse adding-doubling algorithm for a set of optical properties (**μa**, **μ**'s and **g**). Then, the lost light for the diffuse and direct fractions is proportionally subtracted and the total reflectance and transmittance is calculated. The loop is repeated by changing the optical properties until the calculated and the experimentally measured values match within a certain tolerance. When this occurs, the fraction of lost light is calculated by carrying out the Monte Carlo simulation. With the lost light correction, new optical coefficients are sought and the process is repeated again until the changes in the values of the absorption and scattering coefficients are less than 0.01%.

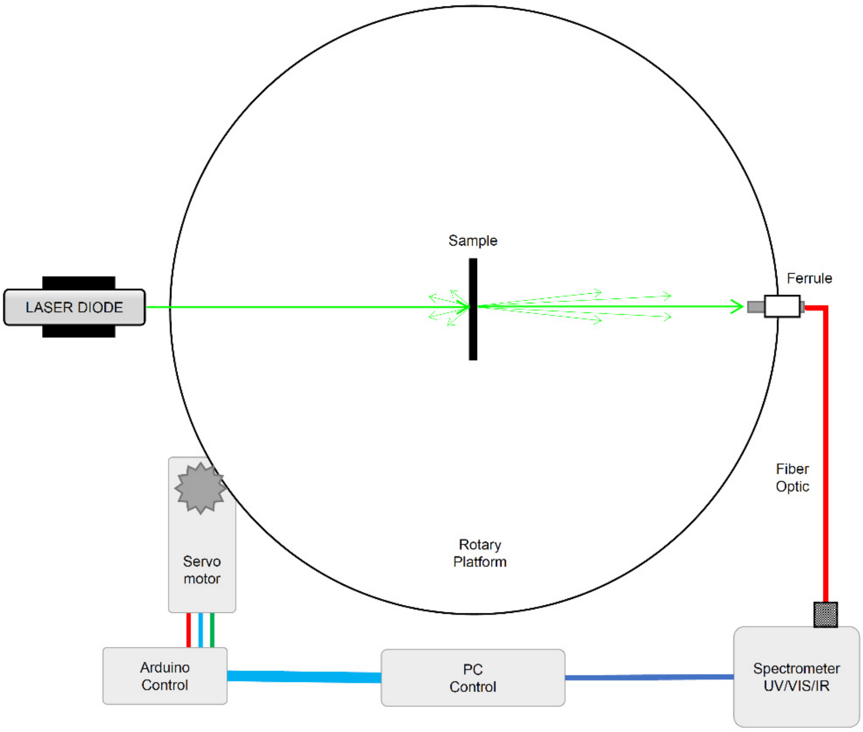

Figure S2. Schematic representation of the experimental setup employed to measure the scattering anisotropy factor.
